# Foreign Bodies in the Eye and Their Removal with the Electro-Magnet

**Published:** 1904-08-27

**Authors:** 


### FOREIGN BODIES IN THE EYE AND THEIR REMOVAL WITH THE ELECTRO-MAGNET.

Marple[1] points out that the majority of foreign bodies which enter the eye consist of iron or steel, upon either of which the electro-magnet will act. The latter instrument may be used (1) as a means of diagnosis, or (2) for removal of the foreign body. For diagnostic purposes the patient is seated in front of the magnet, when he will probably feel pain in the site of the particle of metal, due to the attractive force exerted upon it. If at first there is no such effect the current should alternately be turned off and on, when pain will almost certainly be felt if a particle of iron or steel is present. To remove a foreign body the magnet may be used in one of two ways: (*a*) The old way of introducing into the eye a sound which is connected with the magnet, or (*b*) gradually putting the patient nearer and nearer the source of power, and thus drawing the foreign body into the anterior chamber, whence it may be removed through the wound of entrance, or through an opening made in the periphery of the cornea, into which a small magnetic sound can be thrust. If the foreign body has passed into, or through, a lens which is no longer transparent, there is no reason to trouble about locating it. Where it is not located the tip of the magnet should be placed over the centre of the cornea, when the foreign body may be drawn into the anterior chamber without doing injury to the ciliary body. Where the lens is transparent and the fragment has penetrated the vitreous chamber it must be removed with the least possible injury, and this is more easily done if its position has first been located. Before the Röntgen rays were introduced the situation of a particle was worked out (unless it could be done by the ophthalmoscope) by a siderscope. This consists of a magnetic needle, which becomes more and more deflected the nearer it is to the metal. The instrument introduced by Hirshberg is the most workable form, and it is passed into the different meridians of the eye, and the position of greatest deflection is noted in each. When a foreign body has been located in the vitreous, it may be removed by means of the giant magnet, care being taken in drawing the particle into the anterior chamber to conduct it round and not through the lens, or an incision may be made through the sclerotic, choroid and retina into the vitreous chamber, and a magnet thrust through the opening thus made. The great objection to this latter method is the injury done to the vitreous, and the greater the injury done to the vitreous the greater the likelihood of detachment of the retina. Hirshberg has pointed out, on the other hand, that detachment has often followed removal by the great magnet—further observation is necessary before the better course can be decided on. Where a piece of metal has been located on the retina Hunt recommends that the giant magnet should be used, and that the patient should be told to look to the extreme right or left, then the magnetic pull will transfer the particle to the equatorial region. As soon as pain is felt the current should be cut off so as to avoid the particle penetrating the retina and choroid. Then the tip of the magnet should be transferred to the centre of the cornea and the current turned on again, and thus the piece of metal would be drawn into the anterior chamber. Some hold that such a particle should be left. The objection to this course is that if it does not become encapsuled, even if it is aseptic, it will destroy the eye by setting up siderosis bulbi, a condition described by Bunge as due to the iron going into solution and staining the different media of the eye to a greater or lesser degree, and causing degeneration and even detachment of the retina. When once the process has begun it is too late to remove the foreign body. Double perforation, where the foreign body passes right through the eye and gets lodged in the retro-orbital tissue, need not always be fatal to the eye, if properly treated. Out of 23 such cases where the foreign body was removed, the eye was saved in six, and in one with unimpaired vision. If the particle were left, cyclitis, and perhaps sympathetic infection of the other eye, would follow.

[1] Med. Record, June 25.

